# Individual Distinctiveness in Call Types of Wild Western Female Gorillas

**DOI:** 10.1371/journal.pone.0101940

**Published:** 2014-07-16

**Authors:** Roberta Salmi, Kurt Hammerschmidt, Diane M. Doran-Sheehy

**Affiliations:** 1 Department of Anthropology, University of Georgia, Athens, Georgia, United States of America; 2 Interdepartmental Doctoral Program in Anthropological Sciences, Stony Brook University, Stony Brook, New York, United States of America; 3 Cognitive Ethology Laboratory, German Primate Center, Göttingen, Germany; 4 Department of Anthropology, Stony Brook University, Stony Brook, New York, United States of America; University of Saint-Etienne, France

## Abstract

Individually distinct vocalizations play an important role in animal communication, allowing call recipients to respond differentially based on caller identity. However, which of the many calls in a species' repertoire should have more acoustic variability and be more recognizable is less apparent. One proposed hypothesis is that calls used over long distances should be more distinct because visual cues are not available to identify the caller. An alternative hypothesis proposes that close calls should be more recognizable because of their importance in social interactions. To examine which hypothesis garners more support, the acoustic variation and individual distinctiveness of eight call types of six wild western gorilla (*Gorilla gorilla*) females were investigated. Acoustic recordings of gorilla calls were collected at the Mondika Research Center (Republic of Congo). Acoustic variability was high in all gorilla calls. Similar high inter-individual variation and potential for identity coding (PIC) was found for all call types. Discriminant function analyses confirmed that all call types were individually distinct (although for call types with lowest sample size - hum, grumble and scream - this result cannot be generalized), suggesting that neither the distance at which communication occurs nor the call social function alone can explain the evolution of identity signaling in western gorilla communication.

## Introduction

Advances in the study of animal vocal communication have shown that many species display higher inter- and intra-individual acoustic variability in their vocal signals than previously thought (e.g., birds: [1]–[3]; bats: [4]–[6]; ungulates: [7], [8]; carnivores: [9]–[11]; primates: [12]–[16]). Although it is less clear whether these subtle acoustic differences are always meaningful for the animals, we know that vocal signals have the potential to carry different kinds of information, which include: (a) diverse external events (i.e. predators, food) and behavioral contexts experienced by the callers (birds: [3]; mammals: [17]; primates: [18]); (b) caller internal state (birds: [19]; elephants: [20]; tree shrew: [21]; hyenas: [9]; marmots: [22]; primates: [23], [24]); and (c) caller identity (birds: [25]; koalas: [26]; pandas: [27]; deer: [8]; hyraxes: [28]; hyenas: [9]; primates: [29]–[31]).

Individual differences in vocal signals could reflect both physical and social characteristics. Physical characteristics are those related to morphological differences including age, sex, body size and vocal tract morphology (goats: [32]; hyenas: [9], [33]; deer: [7]; primates: [34]–[37]), whereas social characteristics are those reflecting specific social categories including rank, kinship, and even group membership (birds: [38], [39]; seals: [40]; dolphins: [41], [42]; bats: [5], [43]; primates: [44]–[47]).

Individually distinct vocalizations play an important role in animal communication, since they may reduce the uncertainty of the external world experienced by listeners, allowing them to respond differentially based on caller identity, age, sex, social rank, and group membership. However, there are many questions not yet fully answered regarding animal identity signaling [48]–[50]. First, is inter- and intra- acoustic variability similar across all calls? Second, are all calls in a species' vocal repertoire individually distinct or has selection for individual uniqueness been stronger on some calls than on others? In the latter case, which of the many calls in a species' repertoire should be more individually recognizable? Third, what acoustic characteristics are responsible for generating distinctive voice cues, and are those characteristics the same across call types? Although systematic study of individual distinctiveness and acoustic variability across the species' complete vocal repertoire is needed to understand the adaptive functions they play in the animal's communication systems, few attempts have been made thus far (skuas: [51]; primates: [12], [52], [53]). Instead, the majority of studies have focused on the analysis of only one vocalization at a time (e.g., [14], [15], [54]) or at maximum two (e.g., chimpanzees: [55], [56]; baboons: [50], [57]; putty-nosed monkeys: [58]).

Four non-mutually exclusive hypotheses, based on the distance at which calls are given [59], the social [60], [61] or spatial context [62] in which they are produced, and the direct effects to the nervous system they can elicit in receivers [63], [64], have been proposed to explain the evolution of individually distinct vocal signals and their variation across a species' vocal repertoire. Among non-human primates studies have yielded differing results, supporting two of the hypotheses proposed: the distance communication hypothesis and the social context hypothesis. Under the distance communication hypothesis, calls given or exchanged over long distance are expected to be more distinct among individuals than those given at close distance, since no other cues could be simultaneously used to enable listeners to recognize the identity of the caller [59]. Support for this hypothesis comes from studies conducted in chimpanzees and mouse lemurs, two primate species in which individuals forage commonly alone or in a fission-fusion system. In both species long-distance calls, which are used when individuals are not in visual contact, were more individually distinct than calls exchanged over close distance [12], [55]. In contrast, under the social context hypothesis, calls used in intragroup social interactions at close distance are expected to be more distinct than louder calls directed to a more generalized audience (e.g. the entire group) [60], although some researchers have suggested that threat calls, despite being exchanged at close distances, might be more stereotypic than other close calls ([61] but see [34], [65]). Support for this hypothesis has been found in primate species that are more spatially cohesive. For instance, among red-capped mangabeys' and female Campbell's monkeys' vocalizations, although all calls are individually distinct, those emitted during social/affiliative interactions showed higher acoustic variation and were more individually distinct than either long or alarm calls [52], [53].

Here we test which of these two hypotheses better explains the pattern occurring in the wild western gorilla's (*Gorilla gorilla*) vocal repertoire. We explore whether the acoustic variability differs across female gorilla call types. If gorilla calls are acoustically distinct among individuals, we also determine whether some call types are more distinct than others. We then explore which vocal characteristics are responsible for generating distinctive voice cues, and whether they are the same across call types.

Western gorillas are an appropriate species for this study since: (a) they inhabit the dense tropical rainforest where vocal signals represent an important communicative channel; (b) they live in cohesive, polygynous groups, and, in contrast with chimpanzees, individuals often interact with each other (R. Salmi pers. obs.), and many of their calls are used for regulating these important social interactions [66], [67]; and finally (c) since group members can be separated by over 700m, all age classes need to communicate with dispersed group members using loud broadcast calls (R. Salmi in revision).

**Figure 1:**
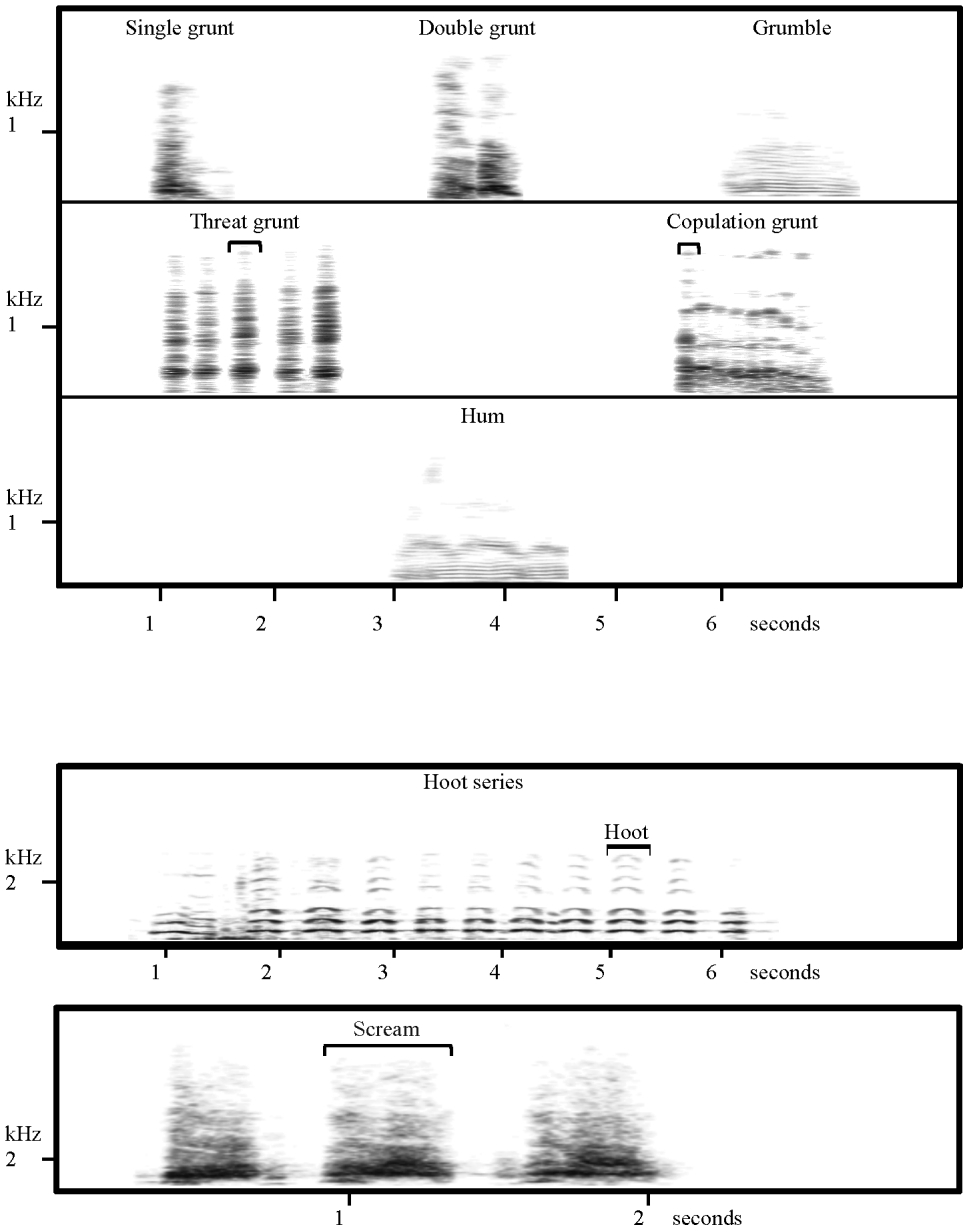
Spectrograms of female gorilla call types. Call types include single grunt (SG), double grunt (DG), grumble (GR), threat grunt (TG), copulation grunt (CG), hum (HM), hoot series (HT), and scream (SC). The call type is equivalent to the call segment, except for TG, CG HT, in which case the call segment is indicated by a bracket.

The species' vocal repertoire has been described recently and includes 17 call types [68]. Since the male body size is more than double than that of the female (170 vs. 71 kg, [69]) and we have data only from one male, to investigate both acoustic variability and individual distinctiveness we examined only calls among adult females. We considered eight call types (Figure 1), which represent the entire vocal repertoire of female western gorillas, except for the play chuckle, a rare call that is difficult to record in the wild [68]. The call types include: soft threat grunts given during within-group aggression; loud screams given during escalated within-group aggression; soft copulation grunts emitted during sexual behavior; single grunts, double grunts, and grumbles, frequent soft calls given during different contexts; hums, given exclusively during foraging at intermediate distance (hereafter referred to as “food call”); and hoot series, long-distance calls given when individuals are separated ([68]; R. Salmi in revision). We measured the same acoustic parameters in all calls and determined both acoustic variability and individual distinctiveness. If the distance at which communication occurs has shaped the acoustic properties of western gorilla vocalizations, we would expect loud calls, such as long-distance calls, to have both higher acoustic variability and individual distinctiveness, and calls exchanged at close distance to show lower values. However, if the social context better explains the variation across call types, we would expect to find calls exchanged at close distance to display higher levels of acoustic variability and individual distinctiveness; food calls (for their indiscriminate target and soft nature) to exhibit intermediate values; and long-distance calls to display lower ones (see Table 1 for a schematic explanation of our predictions).

**Table 1:** Hypotheses and predictions for acoustic variability and individual distinctiveness across western gorilla call types.

*Call*	Distance Hypothesis	Social Hypothesis	
	Predictions		Result
Hoot series	High	Low	High
Hums	Low	Intermediate	High*
Single grunt	Low	High	High
Double grunt	Low	High	High
Grumble	Low	High	High*
Copulation grunt	Low	High	High
Threat grunt	Low	High	High
Scream	Low	High	High*

The last column summarizes the results.

* Not generalizable since p-values generated by cross-validated permutated DFAs did not reach significance.

## Methods

### Ethic statement

This study, based on non-invasive research, followed standard guidelines to prevent disease transmission between observers and animals, and was in compliance with the legal requirements of the country in which it was conducted and the Stony Brook University Animal Care and Use Committee (IACUC numbers: 20071444 and 20091444, 20101444). Permission to conduct research at the site was received from the Ministere de la Économie de Forêt of Congo Republic.

### Subjects and study area

Study subjects included six adult female wild western gorillas from a single group at the Mondika Research Center (02° 21′ 859˝N; 016° 16′ 465˝E), Republic of Congo (for study site description, see [70]). Three of the six females were present during the entire 16-month study period (May-June 2007, April 2009-May 2010), whereas three others were present for shorter periods, due to death and immigration during the study. (Table S1 in File S1).

### Data collection

Data were collected during 2–4 hour focal follows (n = 331) during a total of 749 focal hours. These include an average of 80±6 follows and 174±15 focal hours for four females, and an additional 12 follows and 54 focal hours for a fifth female (Table S1 in File S1). During focal follows, we continuously collected digital recordings of all focal vocalizations, using a portable Marantz recorder (PMD671) and a Sennheiser MKH 416 short shotgun microphone (equivalent noise 13dB, minimal impedance 25Ω, sensitivity in free field, no load (1kHz) 25mV/Pa ± 1dB), handled with a shock mount and protected by a foam windshield (MZW415ANT). Recordings were taken at a sampling frequency of 48 kHz at a distance of 5 meters or less from the focal subject. An additional 62 rare vocalizations were also recorded opportunistically from a sixth female from a comparable distance.

### Data analysis

Using the Avisoft SASLab Pro 5 software (R. Specht, Berlin, Germany), we generated spectrograms (1024-pt FFT length containing 256 samples; overlap 93%) after converting the sampling frequency of the calls depending upon call frequency range (22,050 Hz, 11,025 Hz, or 4000 Hz). Gorilla vocalizations are typically composed of one or more call segments, each of which may be given once or more than once (Figure 1). We use the call segment, rather than the entire vocalization, as the unit for acoustic analysis, following Bezerra et al. [71]. We analyzed all call segments within a sequence for all call types that consisted of repeated call segments of a similar type (i.e., threat grunts, copulation grunts and hoot series), because the segments within a sequence showed high variability. Only independent sequences (separated by at least 1 hr) were considered. Of the 2,360 calls recorded, we selected 681 for acoustic analysis based on the acoustic quality of the recording (i.e. background-noise ratio). We analyzed all eight female call types that make up the female vocal repertoire including the single, double, copulation and threat grunts, grumbles, hums, screams and hoot series. These calls varied in amplitude (soft and loud calls), typical exchange distance (short, medium and long range call distance), whether they were directed to broad or specific audiences, and the context in which they occurred (Table 2) [68]. Average call rates varied between both call types and individuals [68], and therefore the sample sizes available for analysis also vary across individuals and call types (see n in Table 3 for complete description of sample size of each female for each call type). Individuals were excluded from the analysis of a particular call type when sample size was less than four calls. To reduce potential bias and for comparative purposes we used the same number of subjects (typically 3) for the analysis of most call types, varying their identity as needed. All recordings used in this analysis were collected during the 2009–2010-study period, except for those recorded in 2007 for female 6, who died in 2008 (Table S1 in File S1).

**Table 2:** Description of female western gorilla call types, including amplitude (soft or loud), typical distance at which they are exchanged, whether they are given to a broad or specific audience and the contexts in which they are given.

Call type	Soft/Loud	Distance	Directionality	Context
Single grunt^1^	soft	close	broad	maintenance activities (forage, rest, travel)
Double grunt^1^	soft	close	broad	maintenance activities (forage, rest, travel)
Grumble^1^	soft	close	broad	maintenance activities (forage, rest, travel)
Copulation grunt^1^	soft	close	specific/?	sexual intercourse
Threat grunt^1^	soft	close	specific	within-group aggression
Scream^1^	loud	close	specific/?	within-group escalated aggression
Hum^1^	soft	intermediate?	broad	Forage
Hoot series^1^ ^,2^	loud	large	broad	when individuals are separated

1 : Salmi et al. 2013; ^2^: R. Salmi in revision.

**Table 3:** Individual distinctiveness in eight western gorilla call types.

*Call type*	*ID*	*n*	*DFA*	*P_1_*	*C-DFA*	*P_2_*	*Chance*	*F*	*Wilk's λ*	*df*	*χ^2^*	*p-value*	*M*	*Significant parameters*
***Single grunt***		***52***	***75.0***	***0.001***	***75.0***	***0.004***		***1***	***0.36***	***2***	***49.57***	***<0.001***	***1***	***fp1mean***
	F1	16	62.5		62.5		*30.0*							
	F2	25	84.0		84.0		*30.0*							
	F4	11	72.7		72.7		*30.0*							
***Double grunt***		***38***	***86.8***	***0.004***	***84.2***	***0.006***		***2***	***0.33***	***4***	***38.69***	***<0.001***	***2***	***pfmin dfa1maloc***
	F1	26	84.6		84.6		*30.0*							
	F4	8	87.5		87.5		*30.0*							
	F6	4	100		75.0		*30.0*							
***Threat grunt***		***149***	***79.2***	***0.001***	***74.5***	***0.001***		***2***	***0.26***	***18***	***189.59***	***<0.001***	***5***	***dfa2mean diffreq pfmean ampratio duration***
	F1	62	82.3		80.6		*30.0*							
	F3	58	79.3		70.7		*30.0*							
	F5	29	72.4		69.0		*30.0*							
***Cop. grunt***		***59***	***86.4***	***0.001***	***83.1***	***0.001***		***2***	***0.21***	***4***	***88.04***	***<0.001***	***2***	***diffreq, pfmean***
	F1	18	77.8		77.8		*30.0*							
	F4	8	87.5		75.0		*30.0*							
	F5	33	90.9		87.9		*30.0*							
***Grumble***		***24***	***87.5***	***0.036***	***79.2***	***0.701*** *		***2***	***0.13***	***6***	***40.28***	***<0.001***	***2***	***tonality fp1amean***
	F1	6	66.7		66.7		*30.0*							
	F2	12	91.7		75.0		*30.0*							
	F3	6	100		100		*30.0*							
***Hum***		***23***	***78.3***	***0.026***	***78.3***	***0.132*** *		***2***	***0.23***	***4***	***28.30***	***<0.001***	***2***	***noise pfmax***
	F1	14	78.6		78.6		*30.0*							
	F4	4	50		50.0		*30.0*							
	F6	5	100		100		*30.0*							
***Hoot series***		***324***	***78.1***	***0.001***	***77.2***	***0.007***		***1***	***0.67***	***7***	***126.95***	***<0.001***	***2***	***dfa1maloc diffreq***
	F1	209	77.0		76.1		*50.0*							
	F2	115	79.1		79.1		*50.0*							
***Scream***		***12***	***100***	***0.04***	***83.3***	***0.255*** *		***1***	***0.12***	***3***	***18.37***	***<0.001***	***1***	***fp1max***
	F1	7	100		85.7		*50.0*							
	F3	5	100		80.0		*50.0*							

Summary of eight discriminant function analyses, including the identity of the females (F1-6), sample size (n) per female, the % of calls assigned correctly to individual based on discriminant functional analysis (DFA) or cross-validated DFA (C-DFA), relative p-values calculated from pDFAs interactions (P_1_ and P_2_), number of Functions (F), Wilk's λ, Chi square (χ^2^) and p-values, number of linear mixed models (M) and significant parameters after LMM and Hochberg correction.

* In cases with low number of calls a meaningful cross-validation was not possible.

We used the custom software program LMA 2011 [72] to calculate twenty acoustic parameters related to time, frequency, energy, relative amplitude characteristics, and tonality (Table 4) for each call segment. Because we used call segments, as the unit of analysis we could not measure the total call duration, the number of call segments in the call or the silent intervals between them, in contrast to some previous studies (e.g., [52]). We excluded measurements of frequency modulation because it was not a noticeable property in gorilla calls. We also excluded parameters that are greatly influenced by external factors such as start and end call frequency measurements [73].

**Table 4:** Acoustic parameters list. Number, type, name and description of parameters used in the analysis.

Parameters	Description
duration	Duration from beginning to the end of the call [ms]
dfa1mean	Mean value of the frequency at which the first quartile of global energy is reached across all time segments [Hz]
dfa1maloc	Location of the maximum frequency at which the first quartile of global energy is reached across all time segments
dfa2mean	Mean value of the frequency at which the second quartile of global energy is reached across all time segments [Hz]
dfa2maloc	Location of the maximum frequency at which the second quartile of global energy is reached across all time segments
df1max	Maximum value of the first frequency in the call which contains more energy than a particular thresholds in all time segments (DF) [Hz]
df1min	Minimum value of the first frequency in the call which contains more energy than a particular thresholds in all time segments (DF) [Hz]
df1mean	Mean value of the first frequency in the call which contains more energy than a particular thresholds in all time segments (DF) [Hz]
diffmean	Minimum difference between first and second dominant frequency bands (DF) [Hz]
diffreq	Mean number of dominant frequency bands (DF)
ampratio1	Amplitude ratio between first and second dominant frequency bands
fp1max	Max frequency first peak (global frequency peak) [Hz]
fp1mean	Mean frequency first peak [Hz]
fp1amean	Mean amplitude first peak [rel. amplitude]
ranmean	Mean frequency range [Hz]
pfmax	Maximum value in all time segments of the frequency with the highest energy [Hz]
pfmin	Minimum value in all time segments of the frequency with the highest energy [Hz]
pfmean	Mean value across all time segments of the frequency with the highest energy [Hz]
noise	Percentage of noisy time segments [%]
tonality	Percentage of tonal time segments [%]

### Statistical analysis

#### Acoustic variability

We used the twenty call parameters as single acoustic characteristics. For each of them, across all call types, we calculated the inter-individual variation means (MEAN_inter_ calculated over the whole set of calls of a given type) and standard deviation (SD_inter_ calculated over the whole set of calls of a given type), and the intra-individual means (MEAN_intra_ calculated by averaging individual means) and standard deviations (SD_intra_ calculated by averaging the SD of every individual's set of calls and totaling it with the SD calculated over the individual means values). We then computed the coefficients of variation, both inter-individually (CV_inter_  =  100x SD_inter_/MEAN_inter_) and intra-individually (CV_intra_  =  mean of individual CV values; with for each individual, CV  =  100x SD/MEAN) for each parameter. We test whether the inter- and intra-individual coefficients of variation differed among call types using the Friedman test. Separately, we computed for each call type inter- and intra-individually mean coefficients of variation (CV_mean_) averaged over all parameters studied (CV_inter-mean_ and CV_intra-mean_).

#### Individual distinctiveness

We assessed whether call types were individually distinct using two methods. First, for comparative purposes, we considered individual distinctiveness measuring the potential of identity coding (PIC) following Robisson et al. [1], since this method was also used in some previous studies of primate communication [52], [53]. PIC is calculated as CV_inter_/CV_intra_. We consider PIC value of greater than 1 as signifying that the parameter is distinct among individuals and can thus be used for individual recognition, as in previous studies (e.g., [1], [52], [53], [56]). We tested whether calls differed in their PIC values using the Friedman test. For each call we also calculated the overall potential for individual identity coding (overall PIC  =  CV_inter-mean_/CV_intra-mean_) to assess whether some calls display higher values than others.

The second method we used to test for individual distinctiveness in call types was a multilevel analysis of variance, following a two-step procedure [72]. We performed discriminant function analysis (DFA) [74] of the acoustic parameters using the stepwise method and then testing for internal validation of the classification with a cross-validation procedure using the leave-one-out method (U-method) [75]. The comparison of DFA and cross-validated DFA indicates whether the profiles are stable [76], especially in cases in which individual call samples are small (<10 calls). Plots of the distribution of discriminant scores were generated for those cases in which more than one discriminant function was used to distinguish among callers and/or more than two subjects were included in the analysis. We then used linear mixed modeling (LMM) [79] to test whether distinctiveness of call parameters (those which correlated >0.45 with the functions generated by DFA) was significant after controlling for unequal samples and after adjusting for multiple analyses using Hochberg's correction [80]. Additionally, in order to obtain the statistical significance (equivalent to p-value) of the mean effect size [56], [77], we ran permutated DFAs (fitting and validation) using a function written by Roger Mundry in R (version 3.0.1; R Core Team 2013), which is based on the function lda of the R package MASS [78]. This procedure, including 100 random selections and 1000 iterations, allows associating p-values to the observed correct classification rates of both the normal and cross-validated DFAs, given enough cases per individuals. To determine whether some call types were more individually distinct than others, we compared the cross-validated percentages of correct assignment (arcsine transformed) of each female caller for the eight call types and ran a LMM, considering call types as fixed factor and caller identity and number of calls/segments as random factors. All statistical tests have been executed using SPSS 20.0, if not otherwise indicated.

## Results

### Acoustic variability across female calls

**Figure 2:**
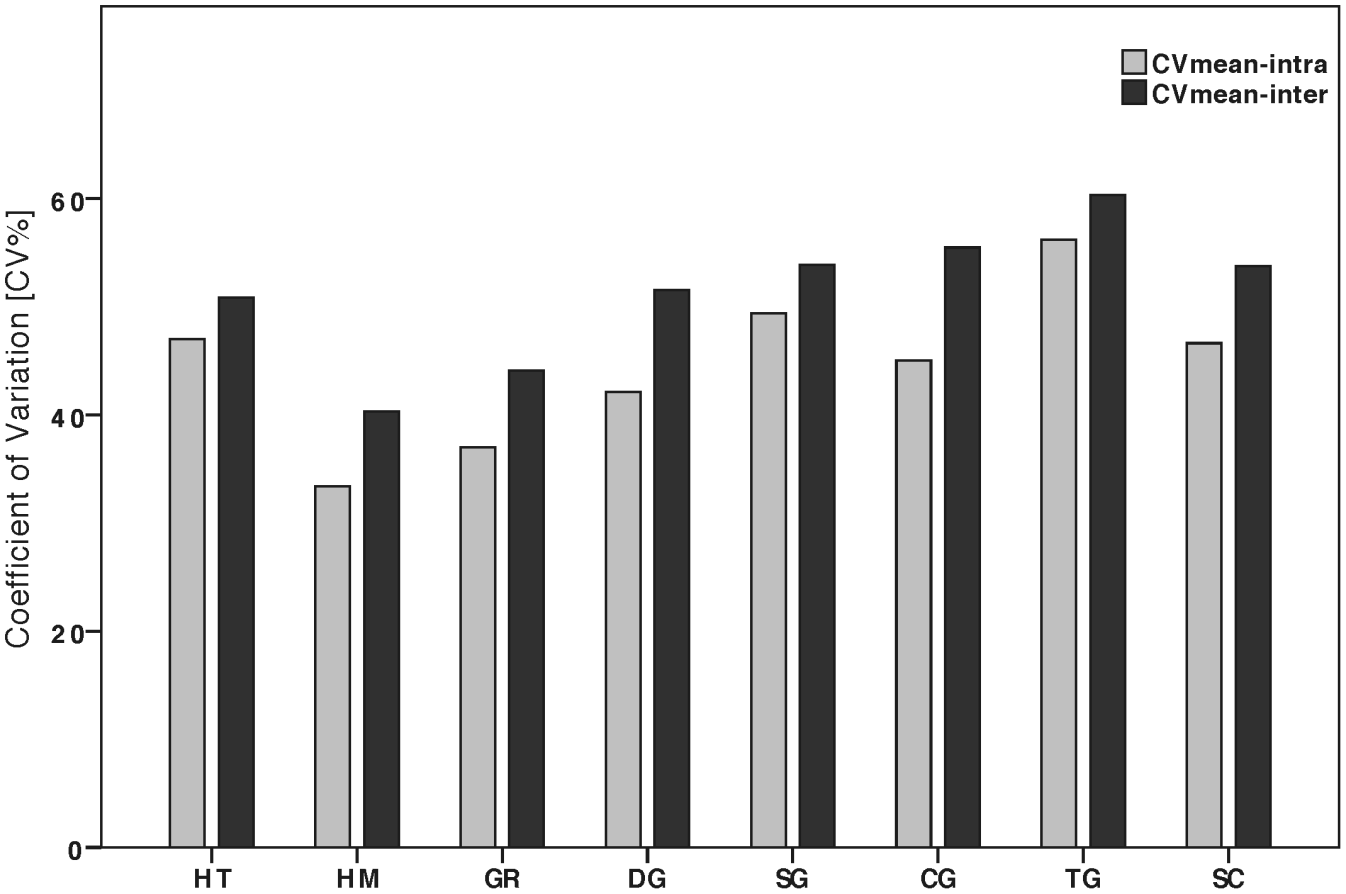
Inter- and intra-individual mean coefficient of variation (CV) for eight gorilla call types. Call types include: hoot series (HT), hum (HM), grumble (GR), double grunt (DG), single grunt (SG), copulation grunt (CG), threat grunt (TG), and scream (SC).

The overall mean coefficients of inter- and intra-individual variation were greater than 40% for most call types (Figure 2; see Table S2 in File S1 for coefficients' values). Intra-individual variation differed significantly across call types (Friedman test on CV_intra_: χ^2^ = 15.57, df = 7, n = 20, p = 0.03; Table S2 in File S1), with CV_intra_ ranging from 33% to 56% in food calls (HM) and threat grunts (TG) respectively (Figure 2). Inter-individual acoustic variation did not differ among calls (Friedman test on CV_inter_ values: χ^2^ = 9.42; df = 7, n = 20, p = 0.22; Table S4 in File S1).

### Individual distinctiveness across female calls

**Figure 3:**
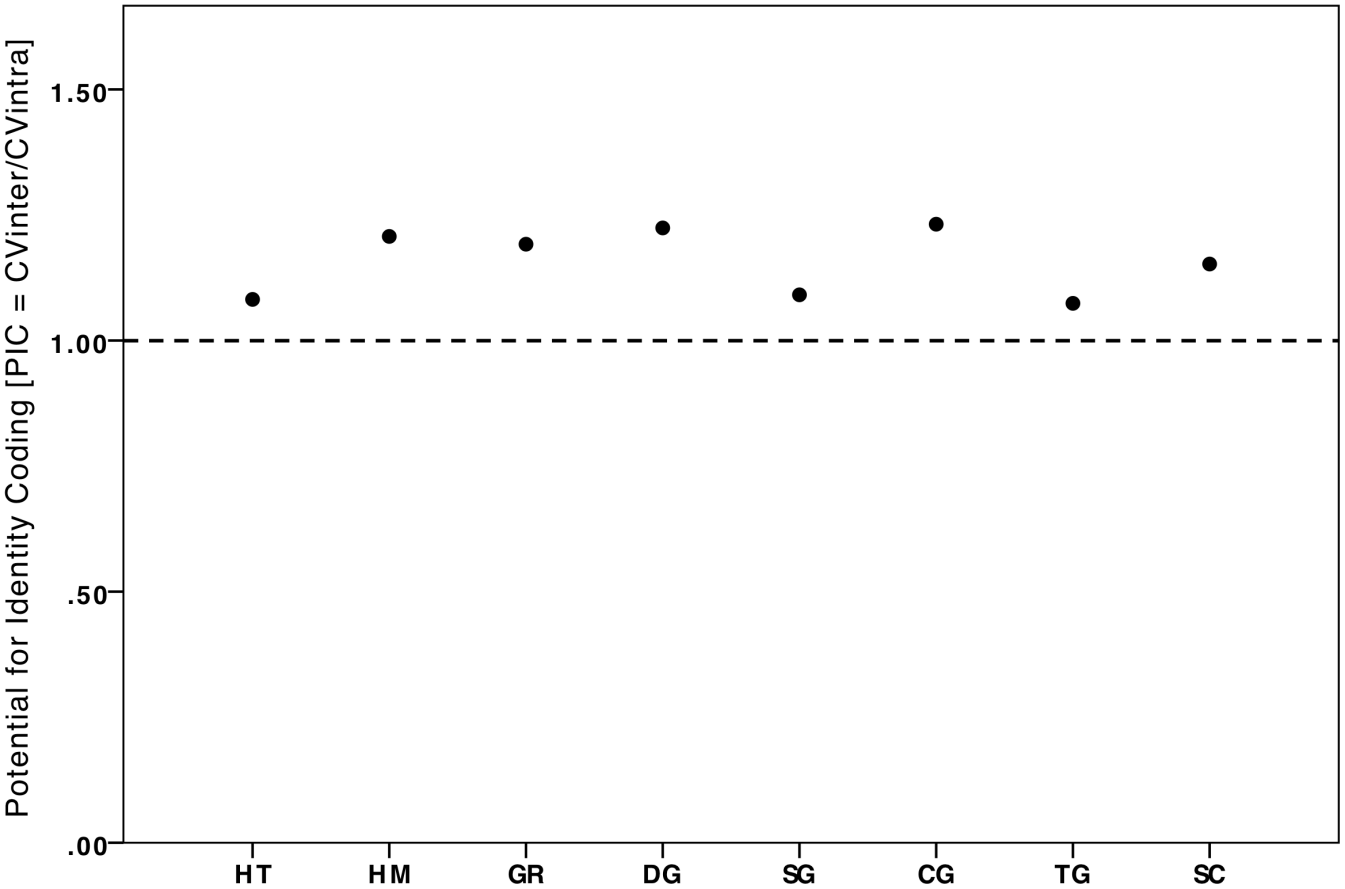
Overall Potential for Identity Coding (PIC) for each of eight call types. The overall PIC (PIC) for each call type is calculated as mean coefficient of inter-individual variation divided by the mean coefficient of intra-individual variation, where each coefficient of variation is averaged over all parameters. Call types include: hoot series (HT), hum (HM), grumble (GR), double grunt (DG), single grunt (SG), copulation grunt (CG), threat grunt (TG), and scream (SC).

The overall potential for identity coding (PIC) values for all call types was >1, and thus all call types displayed individual vocal cues (Figure 3) There was relatively little variation in PIC values across call types (range  = 1.07–1.23, n = 8 call types) and PIC values did not vary significantly among call types (Friedman test: χ^2^ = 7.73, df = 7, n = 20, p = 0.36; Table S3 in File S1). There is no indication that PIC values tended to vary based on call function since the three call types with equally low PIC values (hoot series, single grunts and threat grunts; Figure 3) differed considerably in amplitude (soft versus loud calls), distance at which they were exchanged (close or long range) and in whether they were given in varied or highly specific contexts (Table 2).

**Figure 4:**
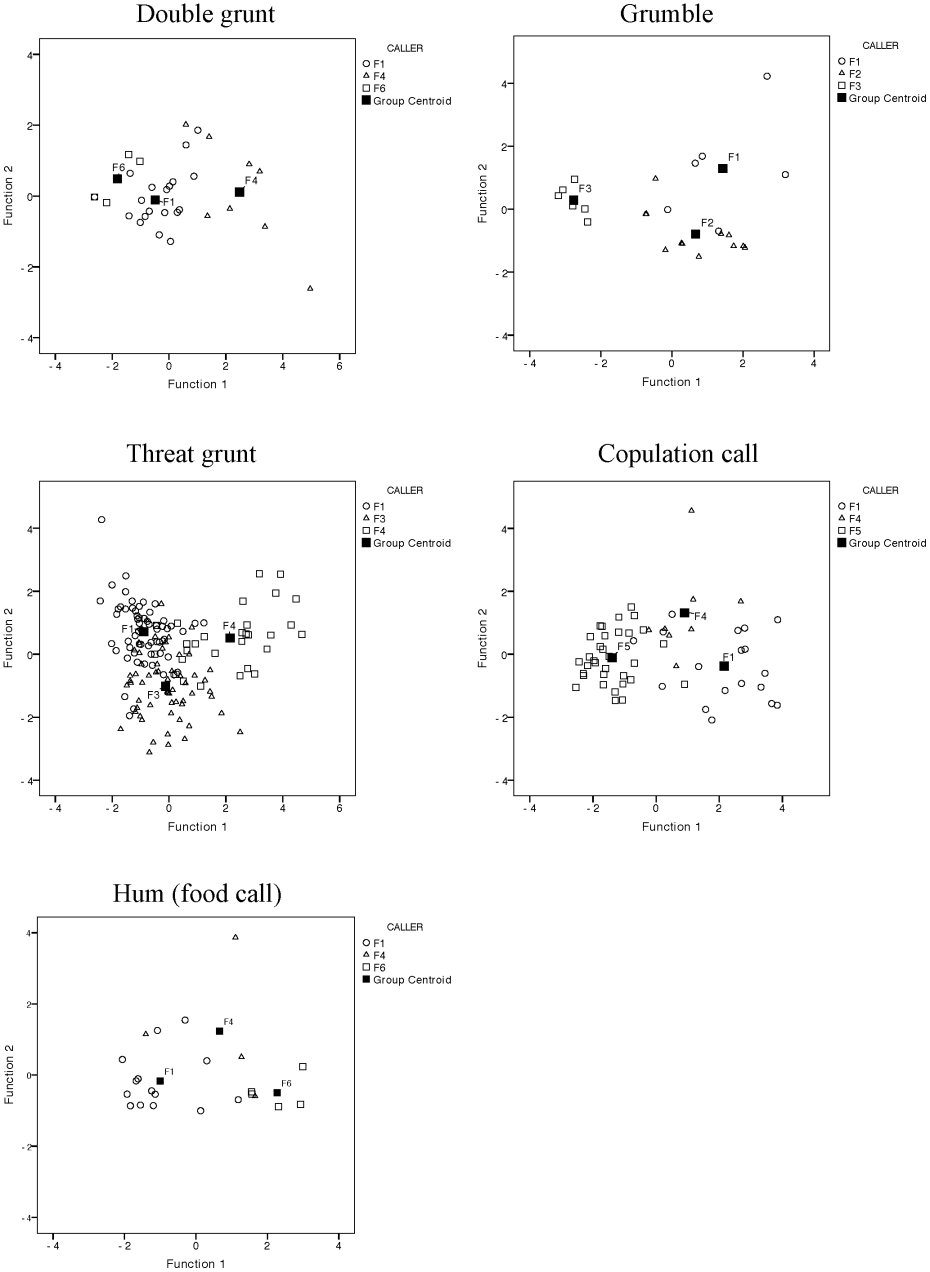
Plots of the two first discriminant functions for five female western gorilla call types based on the DFA of three females. Each individual is indicated with a different symbol.

**Figure 5:**
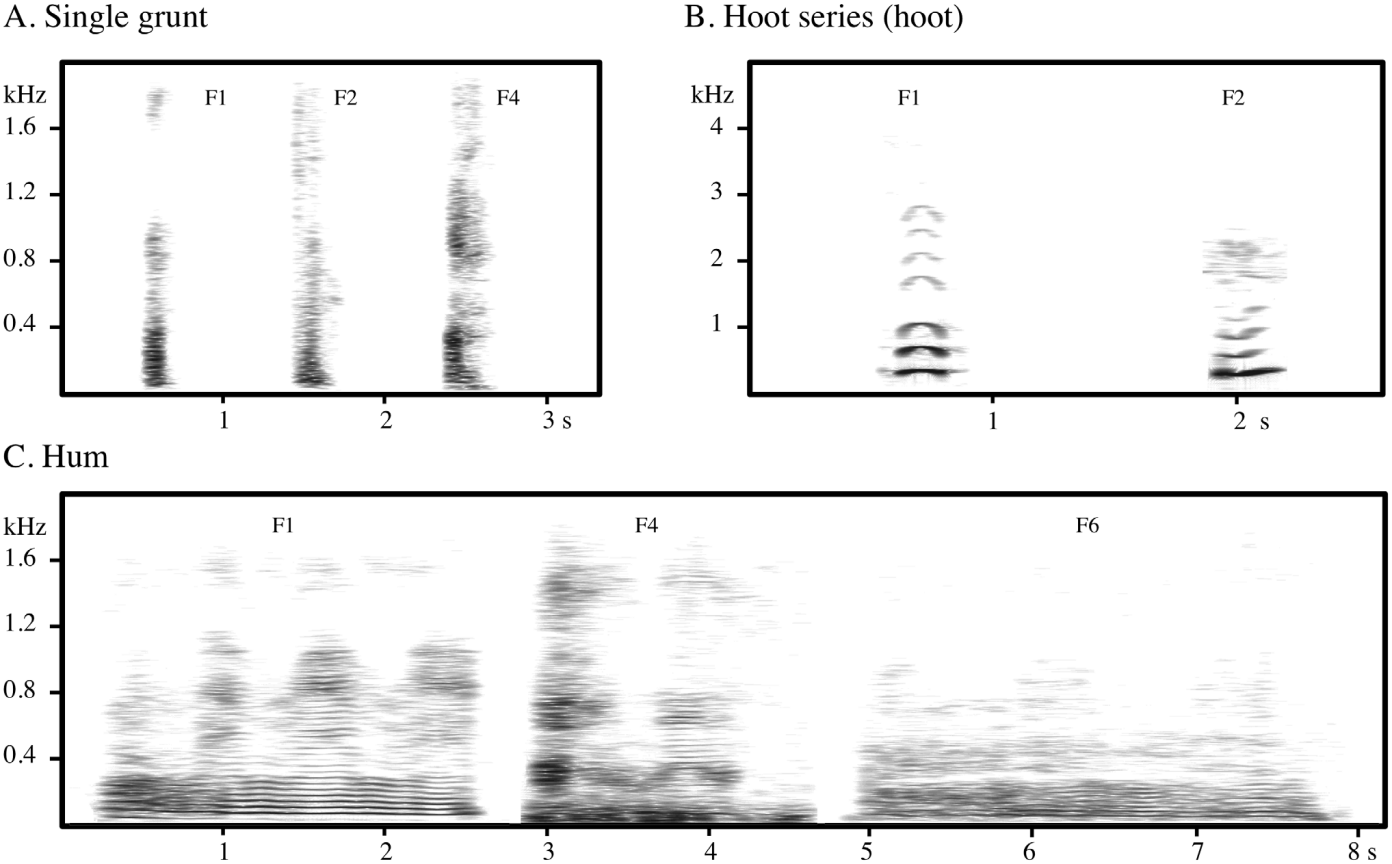
Spectrograms of three call types given by different female gorillas (F1, F2, F4, F6) showing the acoustic variation.

Discriminant function analyses also distinguished among female callers for all eight call types; classification accuracy ranged from 75% in single grunts to 100% in screams (Table 3; cross-validated DFA  = 75 to 84%). The percentage of calls assigned correctly to each subject ranged from 50 to 100% (cross-validated DFA 50 to 100%), which was, in each case, significantly greater than would be expected by chance (i.e. 33% when n = 3 females and 50% when n = 2 females). Plots were generated by the discriminant function analyses for five call types (Figure 4), and in each case females showed a degree of separation, with some gradation in acoustic properties. In each case the DFA and cross-validated DFA produced similar results, indicating that the derived profiles were stable (Table 3). Individual variation in call type can be observed in the spectrograms of the same call type given by different individuals (Figure 5). Using permutated discriminant function analyses (pDFAs) we obtained significant discrimination levels for each DFA (p-values (P_1_) ranging from 0.001 to 0.036; Table 3). The discrimination level for cross-validated p-DFAs (P_2_) was significant for all call types except those with very low numbers of calls (hum: 18 calls; grumble: 18 calls; scream: 10 calls) for which a meaningful permutated cross-validated DFA was not possible. Therefore the results for these three call types cannot be generalized. No call type, however, resulted to be more individually distinct than any other, i.e., cross-validated classification assignments did not differ significantly among the eight call types (LMM controlling for unequal sample size, caller identity and number of individual calls/segments per call type: F_(7, 14)_ = 0.24; p = 0.97).

### Important acoustic parameters

#### From PIC analysis

Based on the PIC analysis, most of the 20 acoustic parameters could be considered useful in distinguishing among callers since they had PIC values >1 (Table S3 in File S1). Five parameters displayed values above 1 for all call types (Table S3: mean frequency range (ranmean), the mean number of dominant frequency bands (diffreq), the mean and the minimum values of the frequency with the highest energy (pfmean and pfmin) and the percentage of noisy time segments (noise). However, the parameters with highest PIC values were not consistent across call types. For example, the minimum difference between first and second dominant frequency bands (diffmean) was very important (PIC>2.0) in distinguishing among individuals in copulation calls, but not in other calls (Table S3 in File S1).

#### From DFA

The discriminant function analysis identified fewer (an average of 2 and range of 1–5) acoustic parameters that were significant in distinguishing among individual callers (Table 3). The set of acoustic parameters that significantly distinguished among callers was different for each call type. For example single grunts were distinguished by the mean frequency of first peak (df1mean), hoots were distinguished by the location of the maximum frequency at which the first quartile of global energy is reached (dfa1maloc) and the mean number of dominant frequency bands (diffreq), and hums were distinguished by the amount of noise in the call and the maximum value of the frequency with the highest energy (pfmax). All parameters used by each DFA to discriminate among callers were still significant after controlling for unequal sample size (LMM) and correcting for multiple analyses (Hochberg's correction) (Table 3; for calls' characteristics see Table S4 in File S1).

No single parameter was significant in all call types (Table 3). However, one parameter, the mean number of dominant frequency bands (diffreq), was used more frequently to distinguish among female callers than any other (i.e. in three of the eight call types, including hoot series, threat grunts and copulation calls). Two other parameters, the location of the global energy in the first quartile of the call (dfa1maloc) and the mean frequency with the highest energy (pfmean) distinguished callers in two call types (i.e., dfa1maloc: double grunt and hoot series; pfmean: threat and copulation grunt; Table 3). No other parameter was significant in more than one call.

## Discussion

We found that all eight call types characterizing the female western gorilla carry important individual cues that could potentially be used by call recipients to distinguish among different callers. This was true regardless of the call amplitude (soft vs. loud) distance at which calls were exchanged (calls used over close and long range), the emotional state of the caller (i.e., calls given when agitated (i.e. cough grunt) versus undisturbed (i.e. grunts)), and the context or lack thereof of calls (i.e. in context-specific calls such as copulation grunts and hoot series versus those given in many contexts such as single and double grunts). Although the PIC analyses, the discriminant function analyses and the normal permuted discriminant function analyses showed that all call types used by western gorilla females were individually distinct, the permutated cross-validated DFAs failed to confirm it for three call types (the hum, the grumble and the scream). Not surprisingly, these calls were those for which we had very low number of calls per individual, which might have strongly lowered the power for a cross-validated method. We find no support in western gorillas for either of the two hypotheses proposed to explain the adaptive function of individual distinctiveness, namely that there is a greater selective pressure for calls to be more individually distinct if they are exchanged over long distances (i.e., the distance communication hypothesis [59]), or used at close range for social communication (i.e., the social communication hypothesis, [60], [61]), in contrast to previous studies in other primate species [12], [52], [53].

This discrepancy may stem, in part, from the more graded nature of apes' vocal systems relative to those of other primates [81]. Graded calls typically exhibit more variability in their acoustic structure than do the more discrete or stereotypic calls typically studied in other primate species ([59], [61], but see [82], [83]). Therefore it was not surprising that the average level of intra-individual variation in each call type was generally higher in western gorillas (33–56%) compared to that of monkey species (red-capped monkeys, *Cercocebus torquatus*: 11–31%, [52]; Campbell monkeys, *Cercopithecus campbelli*: 20–50% [53]). Inter-individual variation was instead intermediate between those of the other two species (red-capped monkeys, *Cercocebus torquatus*: 20–50% [52]; western gorillas, *Gorilla gorilla*: 40 to 60% (this study); Campbell monkeys, *Cercopithecus campbelli*: 45–227% [53]), in which alarm calls were consistently less distinct than other calls. It is possible that the absence of predator alarm calls in western gorillas contributes to the relative similarity of individual distinctiveness since these calls are typically more stereotypic and less variable across individuals than other calls [52], [53].

Although individual acoustic variability and distinctiveness and the potential ability to distinguish among callers may have evolved in many taxa (e.g., birds: [1]–[3]; bats: [4]–[6]; ungulates: [7], [8]; carnivores: [9]–[11]; primates: [12]–[16]), this capacity may be particularly beneficial to primates, because in the majority of primate genera individuals live long lives (i.e., they have slow life histories, [84]), in the company of other group members, with whom they develop long-term relationships. The ability to recognize all types of vocalizations of others would be beneficial because it could potentially allow an individual to monitor the interactions of others while engaged in other maintenance activities (e.g., [85]–[89]), avoid the unseen approaches of higher-ranking individuals, and allow the coordination of group activity and spatial proximity (e.g., [90], [91]) when group members are spatially separated [92], [93]. In western gorillas it might be especially important for silverback males to be able to distinguish among the calls of different female callers, since males are actively involved in protecting group members from predation, harassment, and infanticide [94], [95], serve as peacekeeper in female conflicts [95], [96] and play an important role in group travel and coordination [97]. Therefore, since all female gorilla calls can serve some sort of social function, independently of the distance at which they are exchanged, our results seem to support the social-vocal coevolution of communicative abilities [98]. Additionally, although western gorilla groups are relatively small [99]–[101], individuals are frequently visually separated from each other (R. Salmi in revision), and thus individually distinct calls may be particularly important in keeping individuals in contact with each other, as the “vocal grooming hypothesis” proposed previously [61], [102].

It has also been suggested that individual distinctiveness might simply be the result of chance, emerging from idiosyncrasies and differences in the development of the vocal tract of each individual [50], [103]. However, this explanation has not gained much traction, because it appears too simplistic and unable to account for the level of individual differences found in vocal signals of primates [104], [105].

To date, it continues to remain unclear the exact role that individual acoustic variability plays in animal communication. An important step toward a better understanding of the evolution and the adaptive function of individual vocal cues in animal communication is testing whether these differences are meaningful and used by listeners to distinguish callers. That calls are individually distinct, in fact, does not mean that individuals can recognize the calls of different individuals, and playback studies are needed to this end (e.g., [31], [46], [106]–[108]).

One question raised by this study is whether there is in fact any biological relevance to finding that one call type is more individually distinct than another, if all call types are distinct. In addition, evidence in primate literature suggests that acoustic recognition might not follow the same pattern of call distinctiveness. For example, Kojima et al. [109] demonstrated that although chimpanzee long-distance calls (pant hoots) were more individually distinct than close calls (pant grunts) [55], a female chimpanzee easily distinguished between different callers for both calls, and recognized caller identities even when calls of more than one individual were played simultaneously. This suggests that acoustic recognition of caller identity is dependent upon some individual distinctiveness, but does not necessarily improve with the relative degree of distinctiveness.

Different acoustic parameters (or set of parameters) were responsible for generating individually distinctive voice cues in the different call types. For example, the significant acoustic parameters for distinguishing among individual single grunts did not overlap with those important for double grunts (Table 4). We did not find that the parameter “noise” was significant in distinguishing individuals in many call types, or that “noisy” calls (such as grunts) were less individually distinct than harmonic calls (such as hoot series), as was suggested previously [12]. There was, however, one parameter, the mean number of dominant frequency bands (i.e., diffreq) that contributed to distinguishing three call types (Table 4). This parameter may be important because might reflect differences in individual vocal tract configuration during the vocalization. It has been suggested that this parameter could potentially overestimate individual variation in calls as a result of sampling bias when calls are recorded at different distances to the microphone [72]. However, that is unlikely in this study since western gorillas are terrestrial, most female calls are of low amplitude and therefore calls used in this analysis were all recorded at close sampling distances, typically less than 5 meters.

An interesting question that emerges from the analysis of call individual distinctiveness across an entire species' repertoire is whether the same individual signatures are present across call types. Although our study was not designed to address this specific question, the fact that different sets of parameters were important in distinguishing among callers in each call type does not exclude this possibility. In fact, vocal tract, age, body size, and other anatomical (or social) individual differences are likely to affect similarly most utterances (or at least those with similar acoustic structure). For example, from our results, several acoustic characteristics (e.g., dfa1 mean, df1max, fp1mean, pfmean) of a specific female (F1) have consistently lower values than those of another female (F4) across most call types (Table S4 in File S1). However, since subjects' identity was not maintained constant across call types, future studies with a proper design should test this possibility in more details.

An additional factor that could contribute to differences between our results and previous work is our use of the call segment rather than entire call as the unit of acoustic analysis. The use of call segments for analysis can reduce the effect of emotional state on call acoustic variability, since higher arousal are generally associated with an increase in the length of the entire call and the number of repetitions of call segments within each call, and a decrease in duration of silent time between call segments (e.g., [22], [24]). However its use is likely to have underestimated the degree of individual variation and distinctiveness relative to previous studies, particularly in calls of long duration, such as hoot series, threat and copulation grunts, because it fails to account for differences in the temporal parameters of the entire calls, which have been found to differ significantly among individuals in other species (e.g., [53]). Nonetheless, our method did not biased our results towards any specific hypothesis, since most of the gorilla call types are singularly uttered, and those that are not include call types that are exchanged at close and long distances.

The greatest limitation of the current study is the small sample size, both in the number of individuals and calls per subject. Western gorillas have been notoriously difficult to habituate [110] and as a result few groups are available for study. Additionally, their small average group size (one male, four females and their offspring; [99]–[101]) further limit the availability of subjects within groups. Furthermore, there is a pronounced rank effect on female calling rates which further limits sample sizes of low-ranking females ([68], R. Salmi in revision). Small sample size restricted our ability to control for factors that might contribute to individual distinctiveness, such as, age and arousal level of the caller (which may affect call frequency characteristics; e.g., [111], [112]) or whether the same acoustic parameters would also remain significant in distinguishing callers, if other individuals were sampled. If sample size was larger we might expect some minor differences between different groups, since it has been shown that groups exhibit some subtle acoustic differences [43], [44]. However, overall we would expect similar findings due to the limited structural variation within a vocal type, although we would also expect a greater number of parameters to be useful in distinguishing individuals in larger groups, as shown in previous studies (e.g., [113], [114]). However, in spite of these limitations our study showed high levels of acoustic variation across the entire vocal repertoire of female western gorillas, with all call types being individually distinct. Just as individual voices in humans provide the ability to distinguish and recognize the identity of speakers (reviewed in [115]), even before the development of speech perception [116], [117], it is likely that the same may be true in western gorillas, as well as other non-human primates. We suggest that the study of caller recognition is critical to elucidate the adaptive function of individual vocal cues, and playback experiments can contribute to this goal by revealing whether listeners use these acoustic differences to recognize caller identity and investigating what parameters are important for this end.

## Supporting Information

File S1 **Table S1.** Summary of female data, including age, time spent in the group, number of follows, total sampling hours and number of calls. **Table S2.** Coefficient of intra- (a) and inter-variation (b) of each acoustic parameter and overall values for each call type. **Table S3.** PIC values of 20 acoustic parameters [CV_inter_/CV_intra_] and overall values [CV_mean-inter_/CV_mean-intra_] for each call type. **Table S4.** Acoustic characteristics (mean ± SD) of call types [scream (SC), threat grunt (TG), copulation grunt (CG), single grunt (SG), double grunt (DG), grumble (GR), hum (HM), and hoot series (HT)]. In bold are indicated those parameters used by the DFAs to discriminate among females.
(DOCX)

## References

[1] Robisson P, Aubin T, Bremond JC (1993) Individuality in the voice of the imperor penguin *Aptenodytes forsteri* - Adaptation to noisy environment. Ethology 94: 279–290.

[2] Berg KS, Delgado S, Okawa R, Beissinger SR, Bradbury JW (2011) Contact calls are used for individual mate recognition in free-ranging green-rumped parrotlets, *Forpus passerinus* . Animal Behaviour 81: 241–248.

[3] Evans CS, Evans L (2007) Representational signalling in birds. Biology Letters 3: 8–11. 17443952 10.1098/rsbl.2006.0561 PMC2373811

[4] Arnold BD, Wilkinson GS (2011) Individual specific contact calls of pallid bats (*Antrozous pallidus*) attract conspecifics at roosting sites. Behavioral Ecology and Sociobiology 65: 1581–1593.

[5] Boughman JW (1997) Greater spear-nosed bats give group-distinctive calls. Behavioral Ecology and Sociobiology 40: 61–70.

[6] Carter GG, Logsdon R, Arnold BD, Menchaca A, Medellin RA (2012) Adult vampire bats produce contact calls when isolated: acoustic variation by species, population, colony, and individual. Plos One 7: e38791. 22719947 10.1371/journal.pone.0038791 PMC3375292

[7] Reby D, McComb K, Cargnelutti B, Darwin C, Fitch WT, et al (2005) Red deer stags use formants as assessment cues during intrasexual agonistic interactions. Proceedings of the Royal Society B-Biological Sciences 272: 941–947. 10.1098/rspb.2004.2954 PMC1564087 16024350

[8] Vannoni E, McElligott AG (2007) Individual acoustic variation in fallow deer (*Dama dama*) common and harsh groans: a source-filter theory perspective. Ethology 113: 223–234.

[9] Theis KR, Greene KM, Benson-Amram SR, Holekamp KF (2007) Sources of variation in the long-distance vocalizations of spotted hyenas. Behaviour 144: 557–584.

[10] Jansen D, Cant MA, Manser MB (2012) Segmental concatenation of individual signatures and context cues in banded mongoose (*Mungos mungo*) close calls. Bmc Biology 10.. 10.1186/1741-7007-10-97 PMC3529192 23206242

[11] Manser MB (2001) The acoustic structure of suricates' alarm calls varies with predator type and the level of response urgency. Proceedings of the Royal Society of London Series B-Biological Sciences 268: 2315–2324. 10.1098/rspb.2001.1773 PMC1088882 11703871

[12] Leliveld LMC, Scheumann M, Zimmermann E (2011) Acoustic correlates of individuality in the vocal repertoire of a nocturnal primate (*Microcebus murinus*). Journal of the Acoustical Society of America 129: 2278–2288. 21476683 10.1121/1.3559680

[13] Clay Z, Zuberbühler K (2011) The structure of bonobo copulation calls during reproductive and non-reproductive sex. Ethology 117: 1158–1169.

[14] Fischer J, Hammerschmidt K, Cheney DL, Seyfarth RM (2001) Acoustic features of female chacma baboon barks. Ethology 107: 33–54.

[15] Fischer J, Hammerschmidt K, Cheney DL, Seyfarth RM (2002) Acoustic features of male baboon loud calls: influences of context, age, and individuality. Journal of the Acoustical Society of America 111: 1465–1474. 11931324 10.1121/1.1433807

[16] Hauser MD (1991) Sources of acoustic variation in rhesus macaque (*Macaca mulatta*) vocalizations. Ethology 89: 29–46.

[17] Macedonia JM, Evans CS (1993) Variation among mammalian alarm call systems and the problem of meaning in animal signals. Ethology 93: 177–197.

[18] Seyfarth RM, Cheney DL (2003) Signalers and receivers in animal communication. Annual Review of Psychology 54: 145–173. 10.1146/annurev.psych.54.101601.145121 12359915

[19] Leavesley AJ, Magrath RD (2005) Communicating about danger: urgency alarm calling in a bird. Animal Behaviour 70: 365–373.

[20] Soltis J, Leong K, Savage A (2005) African elephant vocal communication II: rumble variation reflects the individual identity and emotional state of callers. Animal Behaviour 70: 589–599.

[21] Schehka S, Zimmermann E (2009) Acoustic features to arousal and identity in disturbance calls of tree shrews (*Tupaia belangeri*). Behavioural Brain Research 203: 223–231. 19445967 10.1016/j.bbr.2009.05.007

[22] Blumstein DT, Armitage KB (1997) Alarm calling in yellow-bellied marmots .1. The meaning of situationally variable alarm calls. Animal Behaviour 53: 143–171.

[23] Seyfarth RM, Cheney DL (2003) Meaning and emotion in animal vocalizations. In: Ekman P, Campos JJ, Davidson RJ, de Waal FBM, editors. Emotions inside out. New York: New York University Press. pp. 32–55. 10.1196/annals.1280.004 14766619

[24] Lemasson A, Remeuf K, Rossard A, Zimmermann E (2012) Cross-taxa similarities in affect-induced changes of vocal behavior and voice in arboreal monkeys. Plos One 7: e45106. 22984618 10.1371/journal.pone.0045106 PMC3440359

[25] Jouventin P, Aubin T (2002) Acoustic systems are adapted to breeding ecologies: individual recognition in nesting penguins. Animal Behaviour 64: 747–757. 10.1006/anbe.1999.1086 10373249

[26] Charlton BD, Ellis WAH, McKinnon AJ, Brumm J, Nilsson K, et al (2011) Perception of male caller identity in Koalas (*Phascolarctos cinereus*): acoustic analysis and playback experiments. Plos One 6: e20329. 21633499 10.1371/journal.pone.0020329 PMC3102089

[27] Charlton BD, Zhang Z, Snyder RJ (2009) Vocal cues to identity and relatedness in giant pandas (*Ailuropoda melanoleuca*). Journal of the Acoustical Society of America 126: 2721–2732. 19894848 10.1121/1.3224720

[28] Koren L, Geffen E (2011) Individual identity is communicated through multiple pathways in male rock hyrax (*Procavia capensis*) songs. Behavioral Ecology and Sociobiology 65: 675–684.

[29] Cleveland J, Snowdon CT (1982) The complex vocal repertoire of the adult cotton-top tamarin (*Saguinus oedipus oedipus*). Zeitschrift fuer Tierpsychologie 58: 231–270.

[30] Snowdon CT, Cleveland J (1980) Individual recognition of contact calls by pygmy marmosets. Animal Behaviour 28: 717–727.

[31] Miller CT, Thomas AW (2012) Individual recognition during bouts of antiphonal calling in common marmosets. Journal of Comparative Physiology a-Neuroethology Sensory Neural and Behavioral Physiology 198: 337–346. 10.1007/s00359-012-0712-7 PMC3799814 22277952

[32] Briefer E, McElligott AG (2011) Indicators of age, body size and sex in goat kid calls revealed using the source-filter theory. Applied Animal Behaviour Science 133: 175–185.

[33] Mathevon N, Koralek A, Weldele M, Glickman SE, Theunissen FE (2010) What the hyena's laugh tells: sex, age, dominance and individual signature in the giggling call of *Crocuta crocuta* . Bmc Biology 10: 3–16. 10.1186/1472-6785-10-9 PMC2859383 20353550

[34] Bouchet H, Pellier A-S, Blois-Heulin C, Lemasson A (2010) Sex differences in the vocal repertoire of adult red-capped mangabeys (*Cercocebus torquatus*): a multi-level acoustic analysis. American Journal of Primatology 72: 360–375. 20052691 10.1002/ajp.20791

[35] Cheney DL, Seyfarth RM, Fischer J, Beehner JC, Bergman TJ, et al (2004) Factors affecting reproduction and mortality among baboons in the Okavango Delta, Botswana. International Journal of Primatology 25: 401–428.

[36] Pfefferle D, Fischer J (2006) Sounds and size: identification of acoustic variables that reflect body size in hamadryas baboons, *Papio hamadryas* . Animal Behaviour 72: 43–51.

[37] Rendall D, Owren MJ, Weerts E, Hienz RD (2004) Sex differences in the acoustic structure of vowel-like grunt vocalizations in baboons and their perceptual discrimination by baboon listeners. Journal of the Acoustical Society of America 115: 411–421. 14759032 10.1121/1.1635838

[38] Bergman TJ (2010) Experimental evidence for limited vocal recognition in a wild primate: implications for the social complexity hypothesis. Proceedings of the Royal Society B-Biological Sciences 277: 3045–3053. 10.1098/rspb.2010.0580 PMC2982026 20462901

[39] Sharp SP, McGowan A, Wood MJ, Hatchwell BJ (2005) Learned kin recognition cues in a social bird. Nature 434: 1127–1130. 15858573 10.1038/nature03522

[40] Charrier I, Mathevon N, Jouventin P (2003) Vocal signature recognition of mothers by fur seal pups. Animal Behaviour 65: 543–550.

[41] Hawkins ER (2010) Geographic variations in the whistles of bottlenose dolphins (*Tursiops aduncus*) along the east and west coasts of Australia. Journal of the Acoustical Society of America 128: 924–935. 20707463 10.1121/1.3459837

[42] Hoffmann LS, Ferlin E, Fruet PF, Genoves RC, Valdez FP, et al (2012) Whistles of bottlenose dolphins: group repertoires and geographic variations in Brazilian waters. Advances in experimental medicine and biology 730: 141–144. 22278468 10.1007/978-1-4419-7311-5_31

[43] Boughman JW, Wilkinson GS (1998) Greater spear-nosed bats discriminate group mates by vocalizations. Animal Behaviour 55: 1717–1732. 9642014 10.1006/anbe.1997.0721

[44] Crockford C, Herbinger I, Vigilant L, Boesch C (2004) Wild chimpanzees produce group-specific calls: a case for vocal learning? Ethology 110: 221–243.

[45] Digweed SM, Fedigan LM, Rendall D (2007) Who cares who calls? Selective responses to the lost calls of socially dominant group members in the white-faced capuchin (*Cebus capucinus*). American Journal of Primatology 69: 829–835. 17253620 10.1002/ajp.20398

[46] Rendall D, Rodman PS, Emond RE (1996) Vocal recognition of individuals and kin in free-ranging rhesus monkeys. Animal Behaviour 51: 1007–1015.

[47] Weiss DJ, Garibaldi BT, Hauser MD (2001) The production and perception of long calls by cotton-top tamarins (*Saguinus oedipus*): acoustic analyses and playback experiments. Journal of Comparative Psychology 115: 258–271. 11594495 10.1037/0735-7036.115.3.258

[48] Ghazanfar AA (2007) Baboon metaphysics: the evolution of a social mind. Nature 448: 535–536.

[49] Ghazanfar AA, Santos LR (2004) Primate brains in the wild: the sensory bases for social interactions. Nature Reviews Neuroscience 5: 603–616. 15263891 10.1038/nrn1473

[50] Rendall D, Notman H, Owren MJ (2009) Asymmetries in the individual distinctiveness and maternal recognition of infant contact calls and distress screams in baboons. Journal of the Acoustical Society of America 125: 1792–1805. 19275336 10.1121/1.3068453 PMC2736728

[51] Charrier I, Jouventin P, Mathevon N, Aubin T (2001) Individual identity coding depends on call type in the South Polar skua *Catharacta maccormicki* . Polar Biology 24: 378–382.

[52] Bouchet H, Blois-Heulin C, Pellier AS, Zuberbuhler K, Lemasson A (2012) Acoustic variability and individual distinctiveness in the vocal repertoire of red-capped mangabeys (*Cercocebus torquatus*). Journal of Comparative Psychology 126: 45–56. 21875177 10.1037/a0025018

[53] Lemasson A, Hausberger M (2011) Acoustic variability and social significance of calls in female Campbell's monkeys (*Cercopithecus campbelli campbelli*). Journal of the Acoustical Society of America 129: 3341–3352. 21568434 10.1121/1.3569704

[54] Spillmann B, Dunkel LP, van Noordwijk MA, Amda RNA, Lameira AR, et al (2010) Acoustic properties of long calls given by flanged male orang-utans (*Pongo pygmaeus wurmbii*) reflect both individual identity and context. Ethology 116: 385–395.

[55] Mitani JC, GrosLouis J, Macedonia JM (1996) Selection for acoustic individuality within the vocal repertoire of wild chimpanzees. International Journal of Primatology 17: 569–583.

[56] Levrero F, Mathevon N (2013) Vocal signature in wild infant chimpanzees. American Journal of Primatology 75: 324–332. 23229622 10.1002/ajp.22108

[57] Owren MJ, Rendall D (2003) Salience of caller identity in rhesus monkey (*Macaca mulatta*) coos and screams: perceptual experiments with human (*Homo sapiens*) listeners. Journal of Comparative Psychology 117: 380–390. 14717639 10.1037/0735-7036.117.4.380

[58] Price T, Arnold K, Zuberbühler K, Semple S (2009) Pyow but not hack calls of the male putty-nosed monkey (*Cercopithcus nictitans*) convey information about caller identity. Behaviour 146: 871–888.

[59] Marler P (1967) Animal communication signals. Science 157: 769–774. 17842771 10.1126/science.157.3790.769

[60] Snowdon CT, Elowson AM (1997) Social influences on vocal development in New World primates. In: Snowdon CT, Hausberger M, editors. Social influences on vocal development. Cambridge, England: Cambridge University Press. pp. 234–248.

[61] Griebel U, Oller DK (2008) Evolutionary forces favoring communicative flexibility. In: Griebel U, Oller DK, editors. The evolution of communicative creativity: from fixed signals to contextual flexibility. MA: MIT Press. pp. 9–40.

[62] Janik VM, Slater PJB (1998) Context-specific use suggests that bottlenose dolphin signature whistles are cohesion calls. Animal Behaviour 56: 829–838. 9790693 10.1006/anbe.1998.0881

[63] Owren MJ, Rendall D (2001) Sound on the rebound: bringing form and function back to the forefront in understanding nonhuman primate vocal signaling. Evolutionary Anthropology 10: 58–71.

[64] Owren MJ, Seyfarth RM, Cheney DL (1997) The acoustic features of vowel-like grunt calls in chacma baboons (*Papio cynocephalus ursinus*): implications for production processes and functions. Journal of the Acoustical Society of America 101: 2951–2963. 9165741 10.1121/1.418523

[65] Pollard KA (2011) Making the most of alarm signals: the adaptive value of individual discrimination in an alarm context. Behavioral Ecology 22: 93–100.

[66] Harcourt AH, Stewart KJ (1996) Function and meaning of wild gorilla ‘close’ calls. 2. Correlations with rank and relatedness. Behaviour 133: 827–845.

[67] Harcourt AH, Stewart KJ, Hauser M (1993) Functions of wild gorilla close calls 1. Repertoire, context, and interspecific comparison. Behaviour 124: 89–122.

[68] Salmi R, Hammerschmidt K, Doran-Sheehy DM (2013) Western gorilla vocal repertoire and contextual use of vocalizations. Ethology 119: 831–847.

[69] Smith RJ, Jungers WL (1997) Body mass in comparative primatology. Journal of Human Evolution 32: 523–559. 9210017 10.1006/jhev.1996.0122

[70] Doran-Sheehy DM, Greer D, Mongo P, Schwindt D (2004) Impact of ecological and social factors on ranging in western gorillas. American Journal of Primatology 64: 207–222. 15470743 10.1002/ajp.20075

[71] Bezerra BM, Souto A, Jones G (2010) Vocal repertoire of golden-backed uakaris (*Cacajao melanocephalus*): call structure and context. International Journal of Primatology 31: 759–778.

[72] Fischer J, Noser R, Hammerschmidt K (2013) Bioacoustic field research: a primer to acoustic analyses and playback experiments with primates. American Journal of Primatology 75: 643–663. 23592340 10.1002/ajp.22153 PMC3698702

[73] Maciej P, Fischer J, Hammerschmidt K (2011) Transmission characteristics of primate vocalizations: implications for acoustic analyses. Plos One 6: e23015. 21829682 10.1371/journal.pone.0023015 PMC3148239

[74] Klecka W (1980) Discriminant analysis. Beverly Hills, CA: Sage.

[75] Hair JF, Andersen RE, Tatham RL, Black WC (1995) Multivariate data analysis. Englewood Cliffs, NJ: Prentice-Hall International, Inc.

[76] Barros KS, Tokumaru RS, Pedroza JP, Nogueira SSC (2011) Vocal repertoire of captive capybara (*Hydrochoerus hydrochaeris*): structure, context and function. Ethology 117: 83–93.

[77] Mundry R, Sommer C (2007) Discriminant function analysis with nonindependent data: consequences and an alternative. Animal Behaviour 74: 965–976.

[78] Venables WN, Ripley BD (2002) Modern applied statistics with S. 4^th^ Edition. New York: Springer.

[79] West BT, Welch KB, Galecki AT (2006) Linear mixed models: a practical guide using statistical software: Chapman & Hall/CRC.

[80] Hochberg Y (1988) A sharper Bonferroni procedure for multiple tests of significance. Biometrika 75: 800–802.

[81] Marler P (1976) Social organization, communication and graded signals: the chimpanzee and the gorilla. In: Bateson PPG, Hinde RA, editors. Growing points in ethology. Cambridge: Cambridge University Press. pp. 239–280.

[82] Fischer J, Hammerschmidt K (2002) An overview of the Barbary macaque, *Macaca sylvanus*, vocal repertoire. Folia Primatologica 73: 32–45. 10.1159/000060417 12065939

[83] Fischer J, Metz M, Cheney DL, Seyfarth RM (2001) Baboon responses to graded bark variants. Animal Behaviour 61: 925–931.

[84] van Schaik CP, Deaner RO (2003) Life history and cognitive evolution in primates. Animal social complexity: Intelligence, culture, and individualized societies. Cambridge: Harvard Univ Press. pp. 5–25.

[85] Maciej P, Patzelt A, Ndao I, Hammerschmidt K, Fischer J (2013) Social monitoring in a multilevel society: a playback study with male Guinea baboons. Behavioral Ecology and Sociobiology 67: 61–68. 23293423 10.1007/s00265-012-1425-1 PMC3536999

[86] Cheney DL, Seyfarth RM (2007) Baboon metaphysics: the evolution of a social mind. Chicago: Univ Chicago Press. x, 348 pp.

[87] Crockford C, Wittig RM, Seyfarth RM, Cheney DL (2007) Baboons eavesdrop to deduce mating opportunities. Animal Behaviour 73: 885–890.

[88] Slocombe KE, Zuberbühler K (2007) Chimpanzees modify recruitment screams as a function of audience composition. Proceedings of the National Academy of Sciences of the United States of America 104: 17228–17233. 17942683 10.1073/pnas.0706741104 PMC2040427

[89] Wittig RM, Crockford C, Wikberg E, Seyfarth RM, Cheney DL (2007) Kin-mediated reconciliation substitutes for direct reconciliation in female baboons. Proceedings of the Royal Society B-Biological Sciences 274: 1109–1115. 10.1098/rspb.2006.0203 PMC2124468 17301022

[90] Robinson JG (1981) Vocal regulation of inter- and intragroup spacing during boundary encounters in the Titi monkey, *Callicebus moloch* . Primates 22: 161–172.

[91] Palombit RA (1992) A preliminary study of vocal communication in wild long-tailed macaques (*Macaca fascicularis*). II. Potential of calls to regulate intragroup spacing. International Journal of Primatology 13: 183–207.

[92] Leighty KA, Soltis J, Wesolek CM, Savage A (2008) Rumble vocalizations mediate interpartner distance in African elephants, *Loxodonta africana* . Animal Behaviour 76: 1601–1608.

[93] Cheney DL, Seyfarth RM, Palombit R (1996) The function and mechanisms underlying baboon ‘contact’ barks. Animal Behaviour 52: 507–518.

[94] Watts DP (1989) Infanticide in mountain gorillas - New cases and a reconsideration of the evidence. Ethology 81: 1–18.

[95] Harcourt AH, Stewart KJ (2007) Gorilla society: conflict, compromise, and cooperation between the sexes. Chicago: Univ Chicago Press. xviii, 459 pp.

[96] Watts DP (1997) Agonistic interventions in wild mountain gorilla groups. Behaviour 134: 23–57.

[97] Watts DP (2000) Mountain gorilla habitat use strategies and group movements. In: Boinski S, Garber PA, editors. On the move: how and why animals travel in groups. Chicago: Univ Chicago Press. pp. 351–374.

[98] Lemasson A (2011) What can forest guenons “tell” us about the origin of language? In: Vilain A, Schwartz J-L, Abry C, Vauclair J, editors. Primate communication and human language: Vocalisation, gestures, imitation and deixis in humans and non-humans. Amsterdam: John Benjamins. pp. 39–70.

[99] Gatti S, Levrero F, Menard N, Gautier-Hion A (2004) Population and group structure of western lowland gorillas (*Gorilla gorilla gorilla*) at Lokoue, Republic of Congo. American Journal of Primatology 63: 111–123. 15258956 10.1002/ajp.20045

[100] Magliocca F, Querouil S, Gautier-Hion A (1999) Population structure and group composition of western lowland gorillas in north-western Republic of Congo. American Journal of Primatology 48: 1–14. 10326767 10.1002/(SICI)1098-2345(1999)48:1<1::AID-AJP1>3.0.CO;2-2

[101] Parnell RJ (2002) Group size and structure in western lowland gorillas (*Gorilla gorilla gorilla*) at Mbeli Bai, Republic of Congo. American Journal of Primatology 56: 193–206. 11948636 10.1002/ajp.1074

[102] Dunbar R (1996) Grooming, gossip and the evolution of language. London: Faber & Faber. 230 p.

[103] Rendall D, Owren MJ, Rodman PS (1998) The role of vocal tract filtering in identity cueing in rhesus monkey (*Macaca mulatta*) vocalizations. Journal of the Acoustical Society of America 103: 602–614. 9440345 10.1121/1.421104

[104] Riede T, Zuberbühler K (2003) The relationship between acoustic structure and semantic information in Diana monkey alarm vocalization. Journal of the Acoustical Society of America 114: 1132–1142. 12942990 10.1121/1.1580812

[105] Hauser MD (1992) Articulatory and social factors influence the acoustic structure of rhesus monkey vocalizations: A learned mode of production? Journal of the Acoustical Society of America 91: 2175–2179. 1597608 10.1121/1.403676

[106] Cheney DL, Seyfarth RM (1980) Vocal recognition in free-ranging vervet monkeys. Animal Behaviour 28: 362–367.

[107] Fischer J (2004) Emergence of individual recognition in young macaques. Animal Behaviour 67: 655–661.

[108] Fugate JMB, Gouzoules H, Nygaard LC (2008) Recognition of rhesus macaque (*Macaca mulatta*) noisy screams: Evidence from conspecifics and human listeners. American Journal of Primatology 70: 594–604. 18318042 10.1002/ajp.20533

[109] Kojima S, Izumi A, Ceugniet M (2003) Identification of vocalizers by pant hoots, pant grunts and screams in a chimpanzee. Primates 44: 225–230. 12884113 10.1007/s10329-002-0014-8

[110] Doran-Sheehy DM, Derby AM, Greer D, Mongo P (2007) Habituation of western gorillas: the process and factors that influence it. American Journal of Primatology 69: 1354–1369. 17486627 10.1002/ajp.20442

[111] Fichtel C, Hammerschmidt K (2002) Responses of redfronted lemurs to experimentally modified alarm calls: evidence for urgency-based changes in call structure. Ethology 108: 763–777.

[112] Hammerschmidt K, Ansorge V, Fischer J (1994) Age-related variations in the vocal repertoire of Barbary macaques. In: Roeder JJ, Thierry B, Anderson JR, Herrenschmidt N, editors. Current Anthropology, Vol II: Social Development, Learning, and behaviour. Strasbourg: Univ. Louis Pasteur. pp. 287–295.

[113] Hammerschmidt K, Todt D (1995) Individual-differences in vocalizations of young Barbary macaques (*Macaca-sylvanus*) - A multi-parametric analysis to identify critical cues in acoustic signaling. Behaviour 132: 381–399.

[114] Pollard KA, Blumstein DT (2011) Social group size predicts the evolution of individuality. Current Biology 21: 413–417. 21333537 10.1016/j.cub.2011.01.051

[115] Kreiman J (1997) Listening to voices: theory and practice in voice perception research. In: Johnson K, Mullenix J, editors. Talker variability in speech research. New York: Academic Press. pp. 85–108.

[116] Decasper AJ, Fifer WP (1980) Of human bonding - Newborns prefer their mothers voices. Science 208: 1174–1176. 7375928 10.1126/science.7375928

[117] Ockleford EM, Vince MA, Layton C, Reader MR (1988) Responses of neonates to parents and other voices. Early Human Development 18: 27–36. 3234282 10.1016/0378-3782(88)90040-0

