# Supplementary material for: Individual Distinctiveness in Call Types of Wild Western Female Gorillas
Source: PLoS One. 2014 Jul 16;9(7):e101940. doi: 10.1371/journal.pone.0101940 (PMC4100815; doi:10.1371/journal.pone.0101940)
Supplement: File S1 — Table S1. Summary of female data, including age, time spent in the group, number of follows, total sampling hours and number of calls. Table S2. Coefficient of intra- (a) and inter-variation (b) of each acoustic parameter and overall values for each call type. Table S3. PIC values of 20 acoustic parameters [CVinter/CVintra] and overall values [CVmean-inter/CVmean-intra] for each call type. Table S4. Acoustic characteristics (mean ± SD) of call types [scream (SC), threat grunt (TG), copulation grunt (CG), single grunt (SG), double grunt (DG), grumble (GR), hum (HM), and hoot series (HT)]. In bold are indicated those parameters used by the DFAs to discriminate among females. (DOCX) [file pone.0101940.s001.docx]

**Supplemental Material**

Table S1. Summary of female data, including age, time spent in the group, number of follows, total sampling hours and number of calls.

| ***Female ID*** | ***Estimated Age*** | ***Presence in the group*** | ***NFOLLOW*** | ***TSAMPLING (hrs)*** | ***NCALLS*** |
| --- | --- | --- | --- | --- | --- |
| F1 | Mature | Beginning*-present | 84 | 176 | 348 |
| F2 | Mature | Beginning*-present | 86 | 189 | 152 |
| F3 | Mature | Beginning*-present | 75 | 154 | 69 |
| F4 | Young | 2008-present | 74 | 176 | 31 |
| F5 | Young/mature | 2008-2011 | - | - | 62 |
| F6 | Old | Beginning*-Dec 2008 | 12 | 54 | 9 |
| **TOT** | **-** | **-** | **331** | **749** | **681** |

*** Female in the group since the habituation started (Doran et al. 2007).**

**Table S2.** Coefficient of intra- (a) and inter-variation (b) of each acoustic parameter and overall values for each call type.

|  | *Long call (HT)* | | *Food call (HM)* | | *Grumble (GR)* | | *Double grunt (DG)* | | *Single grunt (SG)* | | *Cop. grunt (CG)* | | *Threat call (TG)* | | *Scream (SC)* | |
| --- | --- | --- | --- | --- | --- | --- | --- | --- | --- | --- | --- | --- | --- | --- | --- | --- |
|  | a | b | a | b | a | b | a | b | a | b | a | b | a | b | a | b |
| duration | 92 | 94 | 38 | 45 | 55 | 59 | 35 | 40 | 36 | 50 | 14 | 19 | 5 | 6 | 50 | 48 |
| dfa1mean | 14 | 14 | 16 | 26 | 16 | 16 | 18 | 27 | 22 | 24 | 15 | 19 | 41 | 50 | 29 | 30 |
| dfa1maloc | 70 | 64 | 59 | 72 | 99 | 97 | 93 | 101 | 85 | 83 | 105 | 99 | 92 | 91 | 57 | 60 |
| dfa2mean | 32 | 33 | 25 | 25 | 25 | 23 | 24 | 35 | 32 | 31 | 13 | 12 | 37 | 49 | 25 | 30 |
| dfa2maloc | 67 | 66 | 43 | 66 | 51 | 54 | 115 | 84 | 69 | 70 | 103 | 94 | 47 | 49 | 64 | 72 |
| df1max | 41 | 56 | 46 | 48 | 44 | 47 | 42 | 66 | 42 | 44 | 25 | 41 | 46 | 46 | 31 | 29 |
| df1min | 23 | 22 | 4 | 4 | 5 | 7 | 4 | 6 | 7 | 7 | 26 | 39 | 20 | 21 | 33 | 35 |
| df1mean | 16 | 16 | 16 | 16 | 7 | 10 | 15 | 14 | 10 | 10 | 24 | 43 | 23 | 25 | 36 | 36 |
| diffmean | 81 | 105 | 44 | 42 | 33 | 42 | 52 | 49 | 55 | 60 | 60 | 137 | 56 | 55 | 26 | 25 |
| dffreq | 35 | 37 | 18 | 21 | 27 | 30 | 27 | 31 | 27 | 28 | 26 | 48 | 38 | 45 | 25 | 34 |
| ampratio1 | 94 | 122 | 19 | 22 | 25 | 24 | 26 | 27 | 21 | 26 | 56 | 103 | 35 | 40 | 51 | 75 |
| fp1max | 20 | 21 | 66 | 77 | 74 | 103 | 41 | 47 | 42 | 50 | 24 | 22 | 74 | 73 | 22 | 27 |
| fp1mean | 20 | 21 | 34 | 53 | 35 | 48 | 35 | 39 | 22 | 39 | 27 | 28 | 71 | 68 | 38 | 39 |
| fp1amean | 47 | 45 | 43 | 40 | 45 | 73 | 43 | 35 | 34 | 40 | 34 | 49 | 49 | 50 | 53 | 58 |
| ranmean | 49 | 55 | 27 | 29 | 23 | 32 | 31 | 48 | 38 | 38 | 28 | 41 | 36 | 43 | 41 | 45 |
| pfmax | 25 | 25 | 52 | 67 | 48 | 64 | 65 | 75 | 70 | 71 | 26 | 25 | 61 | 80 | 21 | 25 |
| pfmin | 22 | 23 | 10 | 14 | 15 | 16 | 12 | 21 | 29 | 32 | 35 | 37 | 76 | 97 | 82 | 83 |
| pfmean | 19 | 20 | 27 | 39 | 27 | 30 | 30 | 31 | 26 | 37 | 23 | 26 | 60 | 77 | 35 | 36 |
| noise | 122 | 124 | 27 | 47 | 28 | 42 | 8 | 9 | 13 | 16 | 65 | 85 | 50 | 52 | 24 | 31 |
| tonality | 14 | 54 | 54 | 54 | 58 | 67 | 127 | 248 | 311 | 319 | 170 | 143 | 207 | 190 | 189 | 258 |
| **Overall CV** | **47** | **51** | **33** | **40** | **37** | **44** | **42** | **52** | **49** | **54** | **45** | **56** | **56** | **60** | **47** | **54** |

**Table S3.** PIC values of 20 acoustic parameters [CV_inter_/CV_intra_] and overall values [CV_mean- inter_/CV_mean-intra_] for each call type.

| **Parameters** | **Long call (HT)** | **Food call (HM)** | **Grumble (GR)** | **Double grunt (DG)** | **Single grunt (SG)** | **Cop. call (CG)** | **Threat call (TG)** | **Scream (SC)** |
| --- | --- | --- | --- | --- | --- | --- | --- | --- |
| duration | 1.01 | 1.19 | 1.06 | 1.16 | 1.39 | 1.37 | 1.20 | 0.95 |
| dfa1mean | 1.02 | 1.62 | 0.98 | 1.47 | 1.10 | 1.30 | 1.24 | 1.04 |
| dfa1maloc | 0.92 | 1.22 | 0.98 | 1.09 | 0.97 | 0.94 | 0.99 | 1.06 |
| dfa2mean | 1.05 | 1.01 | 0.92 | 1.44 | 0.97 | 0.91 | 1.31 | 1.22 |
| dfa2maloc | 0.98 | 1.53 | 1.05 | 0.73 | 1.02 | 0.91 | 1.04 | 1.12 |
| df1max | 1.35 | 1.04 | 1.07 | 1.58 | 1.05 | 1.60 | 1.01 | 0.94 |
| df1min | 0.96 | 1.02 | 1.29 | 1.54 | 1.01 | 1.49 | 1.03 | 1.04 |
| df1mean | 1.01 | 1.01 | 1.31 | 0.91 | 0.97 | 1.74 | 1.06 | 1.00 |
| diffmean | 1.31 | 0.96 | 1.26 | 0.94 | 1.09 | 2.27 | 0.97 | 0.95 |
| dffreq | 1.04 | 1.13 | 1.12 | 1.13 | 1.07 | 1.81 | 1.18 | 1.33 |
| ampratio1 | 1.29 | 1.19 | 0.96 | 1.04 | 1.25 | 1.84 | 1.13 | 1.48 |
| fp1max | 1.05 | 1.16 | 1.40 | 1.15 | 1.20 | 0.92 | 0.99 | 1.22 |
| fp1mean | 1.08 | 1.56 | 1.37 | 1.14 | 1.74 | 1.01 | 0.97 | 1.02 |
| fp1amean | 0.97 | 0.93 | 1.63 | 0.81 | 1.17 | 1.45 | 1.02 | 1.10 |
| ranmean | 1.11 | 1.07 | 1.40 | 1.55 | 1.01 | 1.46 | 1.17 | 1.08 |
| pfmax | 1.00 | 1.30 | 1.33 | 1.15 | 1.08 | 0.96 | 1.32 | 1.20 |
| pfmin | 1.07 | 1.31 | 1.05 | 1.67 | 1.10 | 1.06 | 1.29 | 1.01 |
| pfmean | 1.07 | 1.44 | 1.11 | 1.04 | 1.43 | 1.13 | 1.29 | 1.03 |
| noise | 1.02 | 1.70 | 1.53 | 1.17 | 1.28 | 1.30 | 1.04 | 1.26 |
| tonality | 1.03 | 1.01 | 1.15 | 1.95 | 1.03 | 0.84 | 0.92 | 1.37 |
| **Overall PIC** | **1.08** | **1.21** | **1.19** | **1.22** | **1.09** | **1.23** | **1.07** | **1.15** |

**Table S4.** Acoustic characteristics (mean ± SD) of call types [scream (SC), threat grunt (TG), copulation grunt (CG), single grunt (SG), double grunt (DG), grumble (GR), hum (HM), and hoot series (HT)]. In bold are indicated those parameters used by the DFAs to discriminate among females.

| ***CALL*** | ***ID*** | ***duration*** | ***dfa1mean*** | ***dfa1maloc*** | ***dfa2mean*** | ***dfa2maloc*** | ***df1mean*** | ***df1max*** | ***df1min*** | ***diffmean*** | ***dffreq*** | ***ampratio*** |
| --- | --- | --- | --- | --- | --- | --- | --- | --- | --- | --- | --- | --- |
| SC | F1 | 211.7±100 | 1038.6±318 | 0.5±0.4 | 1807.4±563 | 0.5±0.5 | 541.4±227 | **906.1±264** | **246.6±94** | 435.6±100 | 6.8±2.1 | 1.8±1.2 |
|  | F3 | 233.2±124 | 869.8±230 | 0.6±0.2 | 1380.4±258 | 0.6±0.2 | 631.2±188 | **888.8±294** | **319.4±91** | 389.6±117 | 4.6±0.9 | 0.8±0.3 |
| TG | F1 | **199.7±11** | 179.6±66 | 0.2±0.4 | **324.9±101** | 0.7±0.4 | 97.3±20 | 118.5±33 | 79.7±16 | 76.2±40 | **8.5±3.1** | **1.2±0.5** |
|  | F3 | **210.6±11** | 208.9±80 | 0.6±0.4 | **382.8±187** | 0.8±0.3 | 97.8±30 | 136.8±63 | 76.9±17 | 74.3±38 | **9.5±4.7** | **0.9±0.3** |
|  | F4 | **211.0±7** | 337.5±158 | 0.6±0.4 | **645.9±207** | 0.8±0.3 | 104.9±20 | 140.5±91 | 86.2±16 | 79.3±52 | **14.3±3.8** | **1.3±0.3** |
| CG | F1 | 241.3±74 | 175.7±25 | 0.3±0.3 | 290.8±33 | 0.4±0.4 | 92.4±19 | 111.2±30 | 79.2±15 | 59.3±21 | 7.8±1.8 | 0.9±0.2 |
|  | F4 | 220.5±14 | 204.8±34 | 0.4±0.4 | 303.6±52 | 0.4±0.5 | 106.0±16 | 139.4±34 | 80.6±12 | 60.9±12 | **7.3±1.7** | 0.8±0.3 |
|  | F5 | 213.1±11 | 238.8±32 | 0.3±0.3 | 285.3±30 | 0.4±0.4 | 160.6±60 | 230.2±59 | 97.6±44 | 163.6±205 | **3.5±1.2** | 1.3±1.5 |
| SG | F1 | 391.3±126 | 141.6±23 | 0.4±0.4 | 304.4±87 | 0.7±0.4 | 86.5±7 | 136.0±35 | 67.3±2 | 64.1±19 | **7.0±2** | 1.2±0.2 |
|  | F2 | 540.8±296 | 132.7±30 | 0.4±0.4 | 311.6±97 | 0.5±0.5 | 88.0±6 | 151.4±74 | 69.0±4 | 102.1±52 | 5.8±1 | 1.5±0.3 |
|  | F4 | 377.1±80 | 169.6±44 | 0.5±0.5 | 318.6±117 | 0.7±0.4 | 92.6±13 | 135.3±69 | 68.3±7 | 84.6±72 | 7.4±2 | 1.0±0.2 |
| DG | F1 | 599.2±241 | 149.4±48 | **0.3±0.3** | 296.1±117 | 0.5±0.5 | 90.7±11 | 174.3±123 | 67.8±4 | 63.7±23 | 7.1±2 | 1.2±0.3 |
|  | F4 | 472.140 | 157.3±19 | **0.7±0.3** | 269.8±64 | 0.7±0.4 | 95.8±12 | 125.1±14 | 69.5±4 | 55.5±30 | 7.1±1 | 1.0±0.3 |
|  | F6 | 794.0±271 | 147.0±16 | **0.2±0.3** | 292.5±28 | 0.3±0.5 | 103.0±21 | 285.5±124 | 63.0±0 | 100.5±67 | 5.4±2 | 1.1±0.3 |
| GR | F1 | 1593.3±556 | 137.0±24 | 0.4±0.5 | 261.8±57 | 0.5±0.4 | 83.3±3 | 161.8±71 | 69.8±4 | 56.2±9 | 5.8±1.1 | 1.5±0.3 |
|  | F2 | 2026.0±1260 | 120.8±15 | 0.4±0.5 | 289.7±45 | 0.7±0.4 | 87.8±9 | 156.3±94 | 66.2±5 | 62.4±30 | 5.1±1.5 | 1.4±0.3 |
|  | F3 | 1838.0±1269 | 123.5±22 | 0.6±0.4 | 247.0±96 | 1.0±0.1 | 95.7±8 | 195±55 | 63.7±2 | 84.5±30 | 3.8±1.2 | 1.5±0.6 |
| HM | F1 | 1958.3±1020 | 150.9±41 | 0.5±0.4 | 324.2±68 | 0.6±0.3 | 82.7±7 | 144.1±59 | 66.3±2 | 56.4±20 | 6.8±1.2 | 1.4±0.4 |
|  | F4 | 1626.0±498 | 129.3±15 | 0.3±0.2 | 289.3±45 | 0.2±0.0 | 96.8±30 | 222.3±102 | 65.0±2 | 72.7±46 | 5.7±1.1 | 1.3±0.2 |
|  | F6 | 2306.4±699 | 112.4±10 | 0.8±0.4 | 245.6±91 | 0.8±0.4 | 94.0±8 | 205±106 | 64.6±2 | 56.0±19 | 5.1±1.0 | 1.3±0.2 |
| HT | F1 | 235.0±221 | 355.9±52 | **0.5±0.2** | 475.3±174 | 0.5±0.3 | 347.3±55 | 391.2±271 | 312.8±60 | 347.3±55 | **2.0±0.8** | 8.6±10.6 |
|  | F2 | 194.4±177 | 374.3±51 | **0.3±0.2** | 477.9±128 | 0.5±0.3 | 350.6±53 | 399.5±53 | 297.4±79 | 350.6±53 | **2.4±0.8** | 4.8±3.2 |

**Table S4. continued.**

| ***CALL*** | ***ID*** | ***fp1mean*** | ***fp1max*** | ***fp1amean*** | ***ranmean*** | ***pfmean*** | ***pfmax*** | ***pfmin*** | ***noise*** | ***tonality*** |
| --- | --- | --- | --- | --- | --- | --- | --- | --- | --- | --- |
| SC | F1 | 1141.9±450 | **1799.9±154.4** | 549.7±306 | 3017.6±1242 | 1072.4±382 | 1827.7±188 | 268.1±234 | 85.3±35 | 13.1±34 |
|  | F3 | 912.0±334 | **1199.6±429** | 935.8±468 | 2022±844 | 833.6±292 | 1268.8±398 | 448.8±343 | 83.8±16 | 6.0±7 |
| TG | F1 | 175.2±101 | 218.5±114 | 276.1±119 | 843.4±305 | **199.5±105** | 230.2±118 | 141.9±103 | 66.8±33 | 14.8±24 |
|  | F3 | 200.2±138 | 249.5±170 | 261.6±122 | 832.2±406 | **217.0±120** | 259.8±151 | 182.2±118 | 59.4±33 | 10.0±20 |
|  | F4 | 175.4±149 | 269.6±275 | 152.7±86 | 1309.2±320 | **444.5±321** | 547.7±400 | 380.1±340 | 80.9±35 | 8.1±21 |
| CG | F1 | 178.1±62 | 227.8±59 | 297.3±115 | 671.2±200 | **183.8±60** | 234.3±85 | 135.5±66 | 76.4±33 | 14.4±25 |
|  | F4 | 257.3±84 | 288.8±97 | 324.1±58 | 592.1±144 | **285.0±61** | 310.6±84 | 202.1±79 | 84.4±16 | 4.5±10 |
|  | F5 | 258.3±37 | 271.3±34 | 497.9±219 | 371.3±110 | **269.1±38** | 278.5±40 | 259.7±47 | 24.0±32 | 31.8±37 |
| SG | F1 | **92.6±20** | 157.2±58 | 210.0±58 | 607.7±201 | 109.6±35 | 187.0±110 | 71.6±36 | 94.8±13 | 1.9±7 |
|  | F2 | **71.6±14** | 136.0±88 | 253.5±119 | 510.1±187 | 89.7±12 | 161.3±135 | 65.4±9 | 89.6±19 | 5.9±15 |
|  | F4 | **147.01±37** | 233.7±55 | 283.5±75 | 631.1±273 | 159.3±51 | 285.3±192 | 68.7±16 | 98.6±4 | 0.2±0 |
| DG | F1 | 108.5±44 | 202.5±113 | 216.8±67 | 592.8±296 | 121.9±40 | **253.1±203** | 62.2±6 | 94.5±10 | 3.6±8 |
|  | F4 | 154.3±36 | 236.3±41 | 228.6±81 | 457.8±82 | 141.1±30 | **245.5±170** | 81.1±19 | 98.9±2 | 0.0±0 |
|  | F6 | 87.0±35 | 206±105 | 149.3±95 | 358.5±91 | 104.0±37 | **214.3±98** | 49.0±2.3 | 92.3±11 | 5.5±8 |
| GR | F1 | 115.8±66 | 426.7±447 | **329.2±153** | 367.0±48 | 114.0±40 | 337.8±119 | 66.5±5.7 | 51.8±12 | **40.8±11** |
|  | F2 | 65.3±10 | 170.1±104 | **150.1±64** | 306.3±134 | 86.2±13 | 273.9±236 | 58.9±10 | 41.0±19 | **52.3±21** |
|  | F3 | 89.7±29 | 212.7±117 | **74.8±34** | 292.3±32 | 113.8±37 | 235.5±52 | 59.5±12 | 81.7±10 | **5.7±6** |
| HM | F1 | 94.3±54 | 247.9±188 | 228.8±64 | 480.7±136 | 101.2±46 | **194.5±107** | 64.3±4.7 | **36.4±14** | 56.9±14 |
|  | F4 | 74.3±17 | 216.5±156 | 235.3±115 | 475.0±94 | 96.0±16 | **390.5±252** | 57.0±10 | **65.3±20** | 29.0±21 |
|  | F6 | 64.6±13 | 125.2±64 | 128.6±67 | 368.2±117 | 79.4±16 | **168.8±60** | 49.4±3.6 | **81.0±10** | 14.2±9 |
| HT | F1 | 366.6±86 | 398.3±94 | 619.3±257 | 587.7±376 | 368.8±83 | 404.3±100 | 327.2±85 | 17.7±23 | 57.4±33 |
|  | F2 | 368.5±57 | 408.5±70 | 368.5±305 | 624.9±214 | 370.4±56 | 424.6±108 | 335.9±58 | 17.0±20 | 59.7±28 |
